# Fast Synthesis of Multilayer Carbon Nanotubes from Camphor Oil as an Energy Storage Material

**DOI:** 10.1155/2014/691537

**Published:** 2014-09-02

**Authors:** Amin TermehYousefi, Samira Bagheri, Kawasaki Shinji, Jalal Rouhi, Mohamad Rusop Mahmood, Shoichiro Ikeda

**Affiliations:** ^1^ChECA IKohza, Department of Environmental & Green Technology (EGT), Malaysia Japan International Institute of Technology (MJIIT), University Technology Malaysia (UTM), Kuala Lumpur, Malaysia; ^2^Nanotechnology & Catalysis Research Centre (NANOCAT), IPS Building, University of Malaya, 50603 Kuala Lumpur, Malaysia; ^3^Nagoya Institute of Technology, Gokiso-Cho, Showa-Ku, Nagoya 466-8555, Japan; ^4^NANO-SciTech Centre, Institute of Science, Universiti Teknologi MARA (UiTM), Shah Alam, Selangor, Malaysia

## Abstract

Among the wide range of renewable energy sources, the ever-increasing demand for electricity storage represents an emerging challenge. Utilizing carbon nanotubes (CNTs) for energy storage is closely being scrutinized due to the promising performance on top of their extraordinary features. In this work, well-aligned multilayer carbon nanotubes were successfully synthesized on a porous silicon (PSi) substrate in a fast process using renewable natural essential oil via chemical vapor deposition (CVD). Considering the influx of vaporized multilayer vertical carbon nanotubes (MVCNTs) to the PSi, the diameter distribution increased as the flow rate decreased in the reactor. Raman spectroscopy results indicated that the crystalline quality of the carbon nanotubes structure exhibits no major variation despite changes in the flow rate. Fourier transform infrared (FT-IR) spectra confirmed the hexagonal structure of the carbon nanotubes because of the presence of a peak corresponding to the carbon double bond. Field emission scanning electron microscopy (FESEM) images showed multilayer nanotubes, each with different diameters with long and straight multiwall tubes. Moreover, the temperature programmed desorption (TPD) method has been used to analyze the hydrogen storage properties of MVCNTs, which indicates that hydrogen adsorption sites exist on the synthesized multilayer CNTs.

## 1. Introduction

Research and development of energy in the 21st century focused on a wide range of renewable energy sources, due to concerns over fossil fuel and its ever-aggravating impact on global warming, environments, and the crisis of natural resource depletion [1, 2]. Carbon nanotubes have gained significant research interest for their potential applications, such as generating electricity [3] and electricity storage [4], that is, the administrable ability to capture, store, and deliver generated power. The properties of CNTs depend on the arrangement of their graphitic rings and the diameter of their helical structure [5].

CVD is a common CNTs synthesis method based on thermal decomposition of hydrocarbon vapors [6]. The properties of CNTs depend not only on the deposition condition but the starting precursor as well [7]. For various kinds of the deposition process, graphite target is commonly used for the preparation of carbon-based materials [8]. Natural essential oils, a major source of renewable energy, are regarded as promising to the world's thirst for energy [9, 10]. Camphor oil is found in wood of the camphor laurel (*Cinnamomum camphora*), which is a large evergreen tree found in Asia, Dryobalanops aromatica, a giant of the Bornean forests, and some other related trees in the laurel family, notably Ocotea usambarensis. Camphor readily ignites and burns without producing any residue. However, camphor (C_10_H_16_O), which consists of both sp^2^ and sp^3^ carbons, is an attractive new material for carbon-based preparation, since graphite has only sp^2^ carbon [11].

The petroleum-based precursors for synthesis of CNTs have been investigated in detail, and the easy availability of high-grade precursors has resulted in the production and process optimization of different types, structure, dimension, and orientations of CNTs. However, the naturally occurring hydrocarbon precursors have generated some interest because of the possibility of production of CNTs from the bank of hydrocarbons that are being renewably produced by nature in a carbon-neutral manner [12]. So, it becomes important to search for new natural renewable precursors that are easily available and are low in cost, such as essential oils. Of course, it calls for studies that are related to yield and quality of the CNTs being produced and the applications of the resultant CNTs.

Grown MVCNTs arrays with different diameter distribution feeding gas velocity (sccm) have significant effects on CNTs, especially on their diameter distribution [13]. To date, the cause of diameter alterations in single CNTs structures has yet to be clearly understood [14]. Heterostructured multilayer CNTs are fabricated via conventional methods separated by continuous steps, which may be repeated to provide three or more layers of CNTs [15]. Various catalyst formulations and reaction conditions have also been developed to enable the formation of multiple layers of CNTs through the use of appropriate catalysts for different layers [16]. Obtaining a comprehensive understanding of the CNTs growth mechanism is necessary to achieve better control of CNTs growth and design possible nanostructures [17].

Several researchers have reported different types of CNTs based on variations in the parameters in multiple processes [18]; however, to the best of our knowledge, the single step developed in this study is a new method that allows for the control of the diameter of CNTs on PSi. In the present study, MVCNTs were successfully synthesized on a PSi substrate in a one-step process using renewable natural camphor oil via CVD. Moreover, the diameter of CNTs produced through the optimized growth condition is limited by controlling the diffusion of feedstock and also hydrogen storage characteristic of the synthesized multilayer CNTs being analyzed.

## 2. Experimental Procedure

The experimental setup is based on horizontal electronic furnaces used to cover the quartz tube during CNTs fabrication. A mass flow controller was used to adjust the velocity of the carrier gas by means of syringe pump into Psi, which was fabricated via selective doping [19].

Camphor oil as a precursor was mixed with ferrocene and then introduced to the inlet of the quartz tube fitted by the first furnace to release the vaporized CNTs. The reaction temperature was increased to 180°C and maintained for 30 min to ensure that the precursor and catalysts were completely pyrolyzed. Ferrocene decomposes to form the iron catalyst necessary for the experiment, while camphor acts as a carbon source (feedstock) of the substrate in the second furnace. The CVD experiments commenced when the deposition temperature of the second furnace reached the optimal temperature (between 750 and 850°C). The exhaust argon gas in the quartz tube induced movement of the amorphous vaporized carbon into the second furnace by means of a mass flow controller, thereby allowing CNTs growth on the surface of the proposed substrate [20]. To identify the relation between the flow rate and CNTs diameter on the substrate, three flow rates were employed at 1 bar. Argon gas was injected into the inlet of the quartz tube at a maximum velocity of 600 sccm. After 10 min, the velocity was gradually decreased to 400 sccm as its median range of the flow rate. Then, after 20 min, the flow rate was decreased to 200 sccm until complete consumption of the carbon source. After 1 h of reaction time, the reactor was cooled down slowly to room temperature in Ar ambient space after the synthesis.

The CNTs were characterized by FESEM (ZEISS Supra 40VP) operated at 5 kV to evaluate the structure and diameter of the samples. Raman spectra were obtained using micro-Raman spectroscopy (Horiba Jobin Yvon-DU420A-OE-325), with Ar^+^ ions at 514.5 nm to determine the adsorption, desorption, and surface area of the samples. The surface structure of the CNTs was confirmed by a VECTOR33 FT-IR instrument. The chemisorption analyses of the synthesized CNTs were done by TPDRO 1100.

During the TPD analysis, the sample, adequately pretreated, is submitted at an increasing temperature at a constant rate and is swept by an inert gas such as helium. The sample surface desorbs the gas that has been previously chemisorbed, and a suitable detector monitors the process. In the TPD studies, the solid system is previously equilibrated until saturation, with a probe gas in isothermal conditions at a given partial pressure [21].

## 3. Results and Discussion

**Figure 1:**
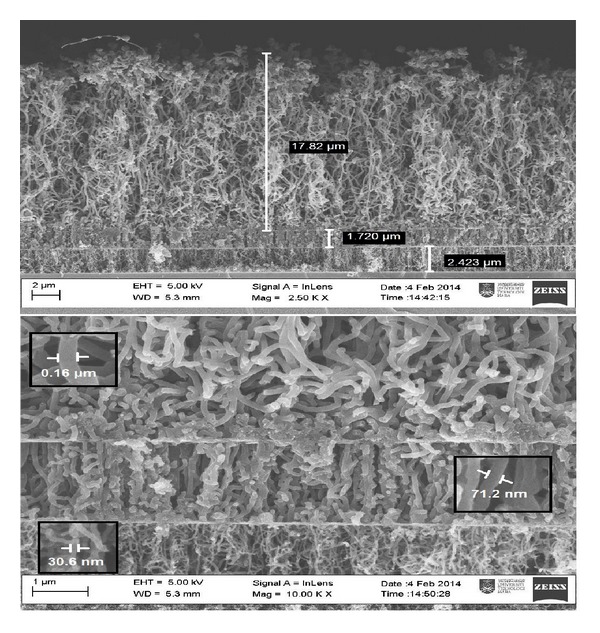
FESEM images of the heterostructured fast synthesized multilayer carbon nanotubes.

As shown in Figure 1, vertically-aligned CNTs with high uniformity and nearly identical diameters were fabricated. FESEM images confirm that well-aligned CNTs with three different diameters were synthesized. As the flow rate decreases, the CNTs gradually thicken [22]. The duration of carrier gas feeding and its flow rate into the reaction zone are key parameters controlling the CNTs diameter [18]. The growth rates of the active ends of the CNTs vary proportionally to the flow rate of argon gas until complete consumption of the carbon source [23]. Alteration of the flow velocity during deposition and limited gas-flow rate control the growth conditions of the CNTs [24].

Considering the influx of vaporized CNTs to the PSi, the diameter distribution increases as the flow rate decreased in the reactor. The quantity of carbons covalently attached to the active end of the tube increased with decreasing flow rates [25]. Various diameters of CNTs are evident during structural transition of the feedstock around the substrate. During the first stage of the experiment, the average diameter of the tubes is approximately 30 nm, and high uniformity is observed. Reducing the flow rate during deposition process changes the diameters of the tubes and produces a central layer with uniformity that is identical to that in the first layer, with an average diameter of 75 nm. The minimum flow rate generates the last layer of multilayer CNTs with the different geometry as the previous layers and average diameter to up to 1 *μ*m; this final layer is obtained by terminating the carbon source.

FESEM images show that variations in flow rate could systematically change the diameter distribution of the CNTs. We propose that at any given flow rate an optimal diameter exists for the CNTs. Varying the carrier gas flow rate during the growth process can be employed to control the growth of CNTs [13].

Given that other parameters, such as carbon feeding rate, temperature, and type of carrier gas, can be altered during CNTs synthesis, our hypothesis also can be developed due to alteration of the other parameters involved with growth process such as multilayer growth of CNTs dependence on temperature [26]. At low temperatures, only small nanoparticles are activated; altering the temperature parallel to the flow rate can change the growth efficiency and aspect ratio of the CNTs [25].

**Figure 2:**
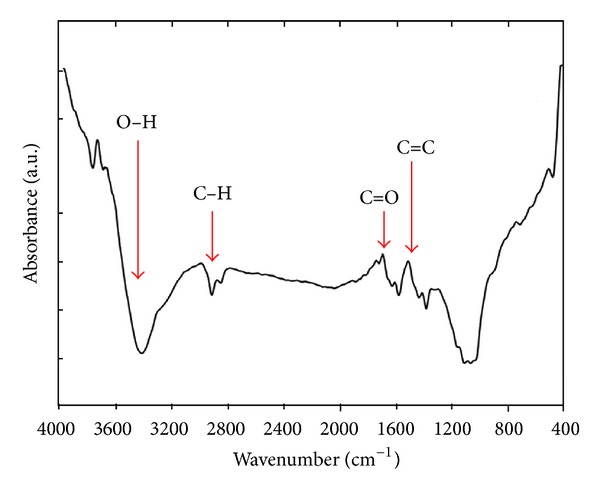
FT-IR spectrum of the fast synthesized multilayer carbon nanotubes.

**Figure 3:**
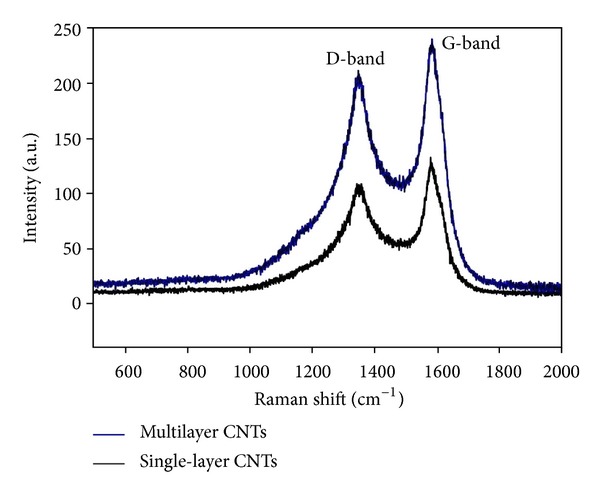
Raman spectra of the fast synthesized multilayer carbon nanotubes.

The FT-IR spectrum (400 cm^−1^ to 4000 cm^−1^) of the fabricated CNTs is shown in Figure 2, and the related peaks are summarized in Table 1. The spectra were recorded using pressed disks of the pure solid powders combined with KBr. Only a small C–C stretch and a peak at 1532 cm^−1^ to 1560 cm^−1^ were observed, which indicates the presence of a carbon double bond (C=C); this finding confirms the hexagonal structure of the CNTs [27].  Figure 3 shows the Raman spectra of the CNTs.

The Raman results have been compared with the growth CNTs in a unique flow rate to ensure the effect of the transitive gas flow on the growth process. Accordingly, two main peaks are found at 1348 cm^−1^ and 1577 cm^−1^; these peaks correspond to the *D* and *G* bands, respectively [28]. The *I*_*D*_/*I*_*G*_ ratio suggests that the crystallinity of the synthesized CNTs under varying flow rates is identical to those grown under a fixed flow rate [29]. The *I*_*D*_/*I*_*G*_ ratio for both types of CNTs is approximately 1.12. As such, the crystalline quality of the CNTs shows no major variation despite changes in flow rate [30].

The growth carbon nanotubes might be a suitable nanostructure for hydrogen storage devices, since for multiwall carbon nanotubes the hydrogen storage capacity is independent of tube's diameter [31]. Furthermore, there are also repulsive forces present between the H and C atoms. This energy tends to become larger as the diameter of tube increases. The potential of nanostructured materials is not only limited to energy storage and conversion devices but also to nanotransistors [32], actuators [33], electron field emission [34], and biological sensing devices [35].

**Figure 4:**
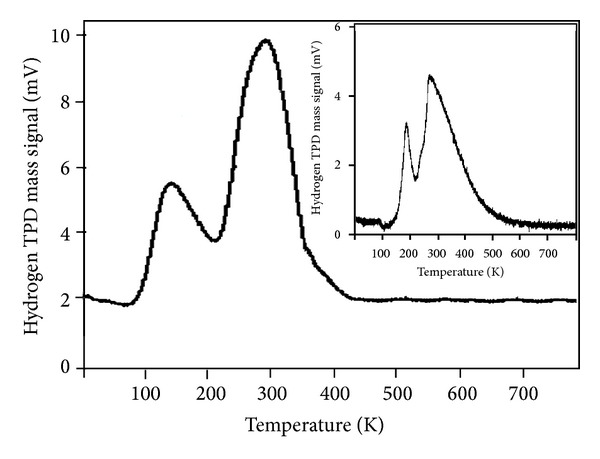
Hydrogen TPD spectra of the fast synthesized multilayer carbon nanotubes. Inset figure represents hydrogen TPD spectra of single layer carbon nanotubes.

The hydrogen adsorption properties of the fast synthesized multilayer CNTs were explored in Figure 4. Accordingly, the sample was degassed to 100°C and exposed to hydrogen at 760 Torr to obtain a TPD spectrum. For comparison, a TDP spectrum of a single layer CNTs also was degassed in the same condition, which was shown in the inset in Figure 4. In both cases, the sample was cooled to ~190 K, while the H_2_ gas was evacuated [36]. These results indicate that unique hydrogen adsorption sites exist on the fast synthesized multilayer CNTs and display a hydrogen adsorption capacity at near ambient conditions that is ~2x greater than that of single layer CNTs. Therefore, conclusively, it can be concluded that multilayer CNTs may also be promising candidates for vehicular hydrogen storage applications [37].

## 4. Conclusion

Well-aligned CNTs with three different diameters have been synthesized by employing different flow rates. FESEM images show that varying the flow rate could systematically change the diameter distribution of the CNTs. Reducing the flow rate during deposition process changes the diameter of the tubes, thereby producing a central layer of CNTs with the same uniformity as that in the first layer. The minimum flow rate generates the last layer of the CNTs structure with the same geometry as the previous layers and high average diameter. FT-IR spectrum indicates the presence of carbon double bonds (C=C), which confirms the integrity of the hexagonal structure of the CNTs. The obtained Raman spectra indicate that the crystallinity of the CNTs structure exhibits no major variation despite changes in flow rate. According to the TPD method for the hydrogen storage properties of MVCNTs, the fast synthesized multilayer CNTs for the hydrogen adsorption capacity at near ambient conditions are ~2x greater than the single-layer CNTs. These results demonstrate a new geometric combination of CNTs based on heterostructured multilayer nanotubes, which can be used in energy storage devices because of their nanosize distribution of carbon nanotubes, accessible surface area, and high stability.

## Figures and Tables

**Table 1:** FT-IR spectroscopy absorption bands of multilayer carbon nanotubes.

Frequency (cm^−1^)	Possible assignment
3424	H-bonded OH groups
2928	C–H bending, stretching
1699	C=O stretching
1540	C=C stretching
